# Genome-Wide Association Mapping of Quantitative Traits in Outbred Mice

**DOI:** 10.1534/g3.111.001792

**Published:** 2012-02-01

**Authors:** Weidong Zhang, Ron Korstanje, Jill Thaisz, Frank Staedtler, Nicole Harttman, Lingfei Xu, Minjie Feng, Liane Yanas, Hyuna Yang, William Valdar, Gary A. Churchill, Keith DiPetrillo

**Affiliations:** * The Jackson Laboratory, Bar Harbor, Maine 04609; † Novartis Institutes for BioMedical Research and Novartis Pharmaceutical Corporation, East Hanover, New Jersey 07936; ‡ Novartis Pharmaceutical Corporation, Basel, Switzerland 4033; § Department of Genetics, University of North Carolina, Chapel Hill, North Carolina 27599

**Keywords:** Mouse Genetic Resource

## Abstract

Recent developments in high-density genotyping and statistical analysis methods that have enabled genome-wide association studies in humans can also be applied to outbred mouse populations. Increased recombination in outbred populations is expected to provide greater mapping resolution than traditional inbred line crosses, improving prospects for identifying the causal genes. We carried out genome-wide association mapping by using 288 mice from a commercially available outbred stock; NMRI mice were genotyped with a high-density single-nucleotide polymorphism array to map loci influencing high-density lipoprotein cholesterol, systolic blood pressure, triglyceride levels, glucose, and urinary albumin-to-creatinine ratios. We found significant associations (*P* < 10^−5^) with high-density lipoprotein cholesterol and identified Apoa2 and Scarb1, both of which have been previously reported, as candidate genes for these associations. Additional suggestive associations (*P* < 10^−3^) identified in this study were also concordant with published quantitative trait loci, suggesting that we are sampling from a limited pool of genetic diversity that has already been well characterized. These findings dampen our enthusiasm for currently available commercial outbred stocks as genetic mapping resources and highlight the need for new outbred populations with greater genetic diversity. Despite the lack of novel associations in the NMRI population, our analysis strategy illustrates the utility of methods that could be applied to genome-wide association studies in humans.

Linkage analysis using crosses between inbred mouse strains is a powerful method for detecting genetic loci underlying quantitative traits (*i.e.*, quantitative trait loci; QTL). However, mapping resolution is typically too coarse to identify the causal genes. Several breeding strategies, including heterogeneous stocks, advanced intercross lines, and the collaborative cross (CC), can produce populations with increased recombination and improved mapping resolution (Flint *et al.* 2005). Recently, both wild-caught and commercial stocks of outbred mice have been proposed as resources for genetic fine mapping (Aldinger *et al.* 2009; Laurie *et al.* 2007; Yalcin *et al.* 2010). Yalcin *et al.* surveyed genetic variation in 66 commercial outbred stocks and identified several with high average minor allele frequency (MAF), low levels of linkage disequilibrium (LD), and absence of population structure as the most promising choices for mapping studies.

Outbred populations have been used previously to refine the location of QTL detected in other mapping studies (Talbot *et al.* 1999). *Rgs2* was identified as the causal gene for anxiety related traits in mice by analyzing 729 outbred MF1 mice with 42 SNP markers spanning a previously mapped QTL interval on Chromosome 1 (Yalcin *et al.* 2004). Outbred populations have also been used for *de novo* QTL mapping similar to genome-wide association (GWA) studies with human populations (Ghazalpour *et al.* 2008; Huang *et al.* 2009; Valdar *et al.* 2006). Valdar *et al.* used an outbred population for genome-wide analysis of cardiovascular disease (CVD) risk factors, but their study included relatively few single nucleotide polymorphisms (SNPs; ∼13,500) compared with the > 100,000 genetic markers typically used in human GWA studies.

Our GWA study using 288 outbred NMRI mice genotyped with the Mouse Diversity Array (Yang *et al.* 2009) was performed with multiple aims in mind. The first aim was to map loci associated with cardiovascular disease risk, including HDL cholesterol, blood pressure, triglyceride levels, glucose, and urinary albumin-to-creatinine ratio (ACR). The second aim was to compare results obtained with different genome scan statistics and permutation strategies. The final goal was to critically assess the utility of the NMRI population as a resource for GWA studies because commercial outbred stocks have been proposed as a novel and potentially powerful resource for genetic mapping (Yalcin *et al.* 2010).

## Materials and Methods

### Animals

Three hundred 4- to 6-week-old, male NMRI mice were purchased from Taconic (Taconic Europe) and individually housed in solid-bottom cages with free access to water and standard rodent chow (cat. no. 8604; Harlan-Teklad). The vivarium was maintained on a 12-hr light/dark cycle (6 am to 6 pm) at 20-24°.

### Phenotyping

Tail-cuff blood pressure was measured in 8-week-old mice using a CODA-6 noninvasive blood pressure monitoring system (Kent Scientific) as previously described (Feng *et al.* 2009). All of the measurements were taken in the afternoon, and values from 100 measurement cycles (20 per day for 5 days) were used to calculate the average systolic blood pressure (SBP), diastolic blood pressure (DPB), mean arterial pressure (MAP), and standard deviation (SD) for each mouse. Any reading >2 SD from the mean for an individual mouse was discarded, and final averages and SD were recalculated. Only mice having a final average SBP calculated from 40 cycles, of 100 cycles maximum, were used for the QTL analyses. Once the mice were 10 weeks of age, spot urine samples were collected daily in the morning for 1 week, and the urine for each mouse was pooled and centrifuged for 5 min at 10,000 rpm. The supernatant was transferred into a clean tube and urinary albumin and creatinine concentrations were measured using a Roche Hitachi 917 Clinical Chemistry Autoanalyzer. A series of mouse albumin standards (Kamiya Biomedical Co., Seattle, WA) was also quantified and the final urinary albumin concentration in each sample was calculated by linear regression from the mouse albumin standards. This method has been shown to accurately quantify albumin concentrations in mouse urine samples (Grindle *et al.* 2006).

Blood samples were obtained from each mouse by submandibular puncture after a 4-hr fast. Plasma samples were frozen at −80° for later measurement of total cholesterol (CHL), high-density lipoprotein cholesterol (HDL-C), triglycerides (TG), and glucose (GLU) using a Beckman Synchron CX5 Chemistry Analyzer. All traits except ACR were approximately normally distributed. The ACR was highly skewed, with many mice having an ACR of zero. We tested a variety of ACR transformations, but all lead to similar conclusions. We report the result for the log-transformed variable logACR = log_e_(ACR + 1). The lipid traits (HDL-C and CHL) and the blood pressure traits (SBP, DBP and MAP) were highly correlated among themselves (*r* > 0.97), and although we analyzed each of the individual traits, results were essentially identical and we report here only our analysis of HDL and SBP, as representative of these groups of traits.

### Genotyping

Genomic DNA was isolated by phenol:chloroform extraction from tail biopsies taken from the 290 NMRI mice that completed the phenotyping protocol, and DNA concentrations were quantified using a Nanodrop spectrophotometer. Samples were genotyped by the Novartis Genomics Factory using the Mouse Diversity Genotyping Array (Yang *et al.* 2009). SNP genotypes were called using a custom software pipeline for this array platform (http://cgd.jax.org/tools/mousedivgeno/). A total of 581,672 SNP genotypes were obtained for each of the 290 animals. Of these, 244,840 SNPs (42%) were polymorphic. Two samples were identified with 99% identity of their SNP genotypes and were removed from subsequent analysis, which was carried out on the remaining 288 mice. We computed the MAF and tested Hardy-Weinberg equilibrium for each polymorphic SNP and retained 103,872 SNPs with MAF > 2%, Hardy-Weinberg equilibrium χ^2^ < 20 and missing values < 40%. The small proportion (1.3%) of missing genotypes that remained were imputed using fastPhase (Scheet and Stephens 2006). Identical SNPs within a 2Mb interval were collapsed resulting in a total of 44,428 unique SNP genotypes that were used in subsequent analyses.

### Population structure

The squared correlation coefficient (*r*^2^) was calculated for all pairs of SNPs within a 50-Mb sliding window across the genome. Locally weighted scatter plot smoothing (LOWESS) was used to compute the median *r*^2^ as a function of distance between pairs of SNPs. The information content of each SNP was computed as the symmetric Shannon entropy, (pln(p) + (1 − p)ln(1 − p)), where p is the MAF of the SNP. In addition to the NMRI population, we computed information content of the full set of 581,672 SNPs genotyped in 79 strains of the BxD recombinant inbred panel and in the eight founder strains of the collaborative cross (Yang *et al.* 2011). A kinship matrix between the individual animals was calculated based on identity by state among the 44,428 SNPs using EMMA (Kang *et al.* 2008). Hierarchical clustering of 288 animals was computed and visualized using the *heatmap* function in R 2.10.0 (R Development Core Team 2009).

### Association mapping

We carried out single-locus association genome scans on diallelic SNPs using each of three statistical tests. We computed a linear trend test (1df) by regressing the trait on genotype scores −1, 0, 1 corresponding to AA, AB, and BB genotypes, respectively. The trend test assumes additive genetic effects at a locus. We also conducted analysis of variance (ANOVA; 2df) that allows for both additive and dominant effects at a locus. Finally we used the EMMA software to fit a mixed linear model at each SNP (described in detail by Kang *et al.* 2008). EMMA accounts for population structure by modeling the variance-covariance matrix as a function of this estimated kinship matrix. The genetic and residual variance components, σ_g_ and σ_e_, are estimated using an efficient implementation of restricted maximum likelihood in EMMA. A linear trend test is computed for each SNP genome-wide and a –log(p) score is reported. Fixed effect covariates can be included in genome scans using any of these statistical tests, which enabled implementation of conditional scans and forward stepwise search by including previously selected marker genotypes as covariates.

### Genome-wide significance thresholds

To assess genome-wide significance of the association statistics, we used a novel simulation technique. We transformed each phenotype using van der Waerden's scores (Conover 1999) and estimated genetic and residual variances of the transformed data for each trait using EMMA. For each phenotype, we simulated 288 trait values by sampling from a multivariate normal distribution using the *mvrnorm* function in R with covariance matrix defined by the estimated kinship. We then replaced the simulated values with observed values in a manner that preserved the rank order of the simulated values. Specifically, we computed the ranks of the original and of the simulated data. The original data value with rank *k* is then reassigned to the individual with *k*th rank among the simulated data. In this way, we obtain a permutation of the original data that retains the correlation structure implied by the kinship matrix. We performed a genome scan using the permuted trait values and recorded the largest –log(*p*) scores. This was repeated 100 times. We fitted a generalized extreme value distribution to these scores and derived significance thresholds from the quantiles of this distribution (Knijnenburg *et al.* 2009).

We also used a free data permutation analysis for each phenotype (Churchill and Doerge 1994). The validity of this simple permutation test is based on an assumption that the individuals in the study are exchangeable. This assumption is not valid when there are different degrees of familial relationship present in the study population; however, we used the simple free permutation as a reference point to assess the impact of applying a more complex, but justifiable, procedure in the context of this study.

### Multilocus analysis

Complex traits are influenced by variation at multiple loci and we can often obtain more realistic estimates of effect sizes and significance by considering the simultaneous effects of multiple loci. We used forward stepwise regression with bootstrap resampling (Valdar *et al.* 2009) to develop multi-locus models for each trait. We first created 100 resampled data sets consisting of trait and genotype values from 288 animals that are sampled at random from the original data with replacement. We performed forward stepwise regression on each resampled data set to obtain a multi-locus regression model with 20 SNPs. The number 20 is arbitrary. All that is required is to ensure that the number of SNPs in the regression model is more than the number that could significantly influence the trait. We calculated the resample model inclusion probabilities (RMIP) for each SNP m as

$$RMIP_{m}=\frac{1}{R}\sum_{r=1}^{R}i_{rm}$$

where *R* = 100 is the number of resampled data sets *i_rm_* = 1 if at least one SNP within ± w Mb of SNP *m* was included in the model of sample *r,* otherwise *i_rm_* = 0. We varied the window size w from ±0.5 Mb to ±4 Mb.

### Precision analysis

To assess the genome-wide average precision of mapping in this population, we performed a simulation study using the observed genotype data. We randomly sampled a SNP from the genome and simulated trait values assuming that this SNP was the causal locus. We simulated an effect size corresponding to the same percentage of total variance explained as the HDL QTL on Chromosome 1 (reported in the section *QTL mapping,* below). Trait values were sampled from a multivariate normal distribution using *mvrnorm* in R with correlation structure defined by the kinship matrix and the genetic and residual variances were as estimated for HDL. We removed the selected SNP from the data and performed a genome scan with EMMA. The distance between the SNP with highest –log(p) and the target SNP was recorded. In cases where a group of adjacent SNPs had tied *P* values, we computed the median distance between the location of association peak and the target SNP. We repeated this process 1000 times, and computed the distribution of distances from the peak to the target SNP. A small proportion of association peaks were observed on different chromosomes from the target SNP. These events were recorded but were excluded from the distance calculation.

## Results

### Genetic parameters of the NMRI population

**Figure 1:**
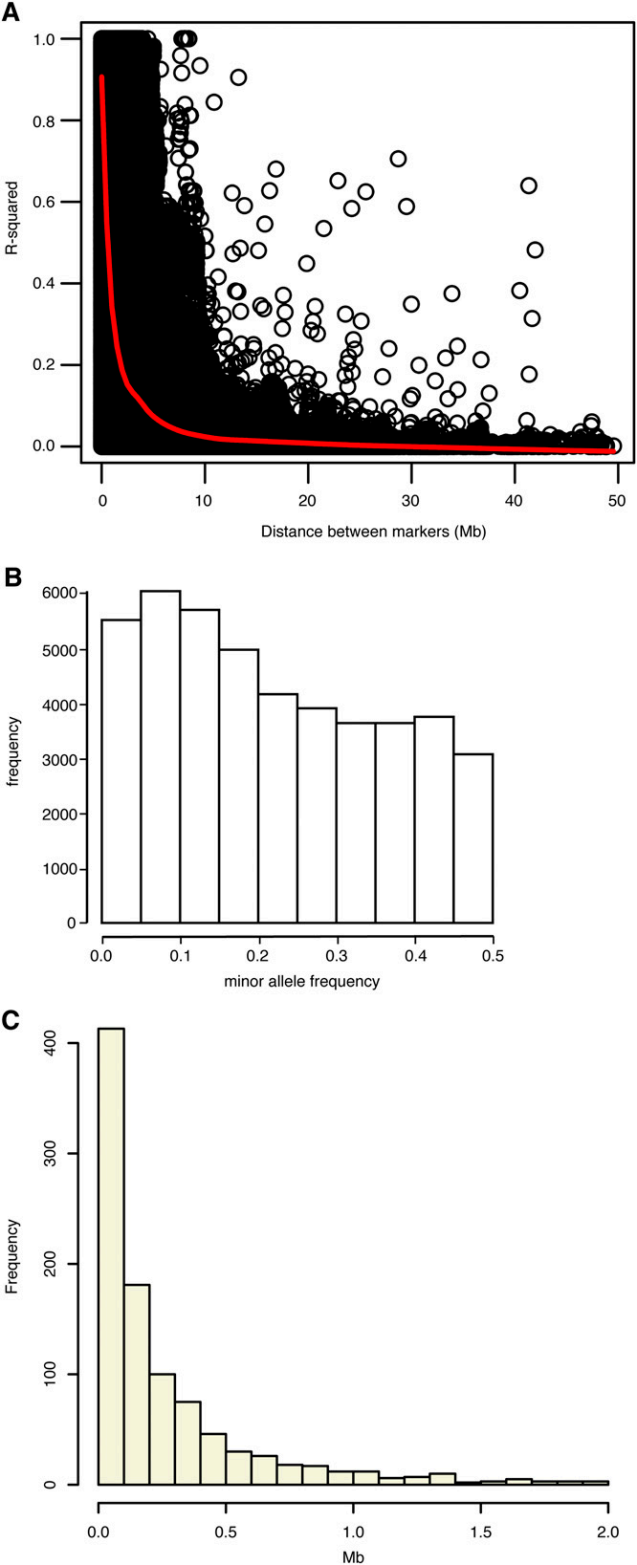
(A) LD between all pairs of makers within a 50-Mb sliding window is computed using the squared correlation coefficient. The red line indicates the local median value of *r*^2^. (B) Distribution of the MAFs of 44,428 SNP markers used in this study. (C) Distribution of the distance between peak marker and location of the causal SNP in 1000 simulated genome scans.

We used SNP genotypes to characterize the genomes of 288 NMRI mice to assess their suitability as a mapping population (the final dataset is available at http://cgd.jax.org/datasets/datasets.shtml). The squared correlation coefficient (*r*^2^) between SNPs was used as a measure of LD. We observed a sharp decay of LD with increasing physical distance between SNPs (Figure 1A). The LD decay radius, defined as the physical distance at which *r*^2^ falls below 0.5, was 1.2 Mb. The distribution of MAFs was broad with 22,205 SNPs having MAF > 0.2 (Figure 1B). The average MAF was 0.23, excluding 336,832 invariant SNPs and 128,923 SNPs with MAF < 0.02. These values for the LD decay radius and average MAF are comparable with other NMRI cohorts, a finding that suggests that NMRI would be a good choice for association mapping (Yalcin *et al.* 2010).

Population structure due to stratification or to family relationships within a sample can adversely affect mapping studies if not accounted for in the analysis (Cheng *et al.* 2010). We assessed the degree of structure in our NMRI population using hierarchical clustering of 288 animals based on the kinship matrix. We observed a few small groups of related animals, but there appeared to be no major structure in our population (supporting information, Figure S1). The mean percentage of identical SNPs (of the 44K filtered set) between any pair of animals was 74%, with a range from 69 to 91%.

Mapping resolution was assessed using simulations as describe in *Materials and Methods*. The maximum –log(*p*) occurred on the same chromosome as the simulated QTL in 99.3% of simulations. When the peak was on the same chromosome it was within 1.34 Mb in 95% of simulations, within 0.52 Mb in 80% of simulations, and within 0.17 Mb in 50% of simulations (Figure 1C). The long right tail of the distribution indicates that mapping resolution was low in some regions of the genome.

The size and distribution of LD blocks was assessed visually using Haploview (Barrett *et al.* 2005) (Figure S2, A and B). LD blocks are larger in our NMRI sample than is usually seen in a human population sample. There are also substantial regions of long-range LD, which is typical of laboratory mouse populations (Petkov *et al.* 2005) and could potentially create spurious associations at SNP markers that are distant from the causal polymorphism (Dickson *et al.* 2010).

**Figure 2:**
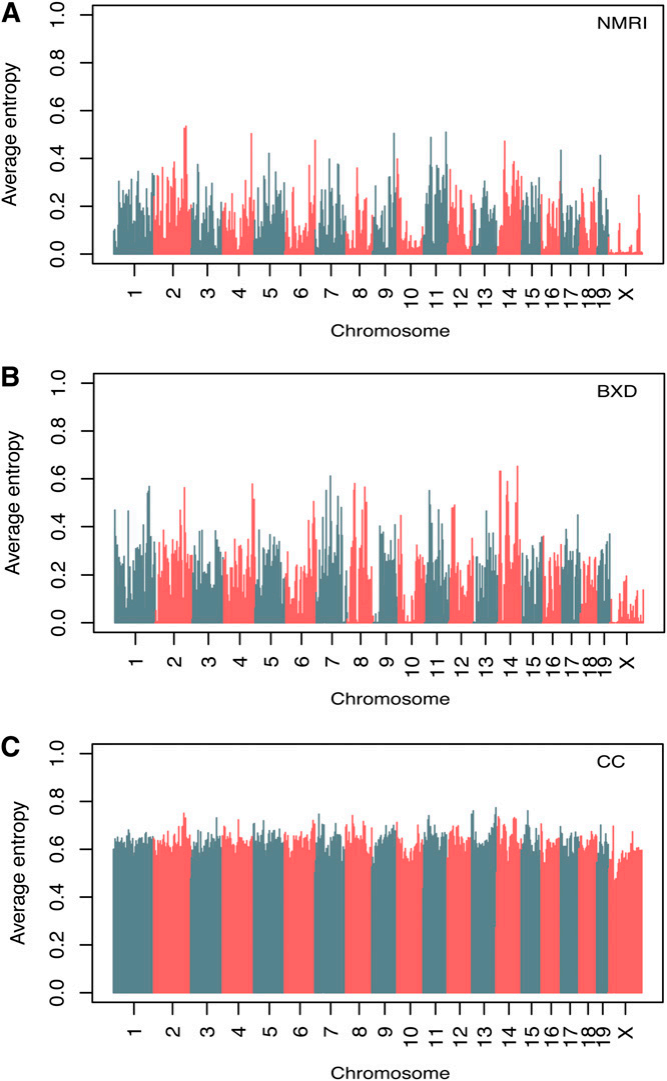
SNP information content. The average Shannon entropy in of SNPs in 4-Mb windows across the genome is shown for NMRI (A), 79 BxD recombinant inbred strains (B), and the eight founder strains of collaborative founder strains (C).

To assess the genetic diversity of NMRI relative to other mouse populations, we computed the mean information content (Shannon entropy) for all 581K SNP markers in our NMRI population, in 79 BxD recombinant inbred strains, and in the eight founder strains of the Collaborative Cross (CC) (Collaborative Cross Consortium 2012). The average entropies for all SNP markers in NMRI, BxD, and CC were 0.10, 0.14, and 0.43, respectively. We computed average information content in 4-Mb windows across the genome in each of these populations to assess local variation in informative SNPs (Figure 2). A number of regions of low diversity were identified in the NMRI and BxD populations, for example on Chromosome 10, consistent with previous reports of the distribution of genetic diversity in classical inbred mouse strains (Yang *et al.* 2007). The distribution of informative SNPs in the CC population is uniformly high.

### Genome-wide significance thresholds

We used two methods to assess significance thresholds for GWA mapping, simulation and unrestricted permutation. We applied these to each of three methods for measuring association: the trend test, ANOVA test, and mixed model. The estimated genome-wide significance thresholds for glucose, HDL cholesterol, systolic blood pressure, and triglycerides were similar across all of these combinations. Values ranged from 5.12 to 5.90, but no single method or trait was consistently higher or lower than another (Table 1). The exception was logACR, which was skewed compared with the other traits (Figure S3). The genome-wide 5% threshold for logACR ranged from 7.76 to 7.79 for EMMA and the simple trend test, but the ANOVA thresholds were substantially greater (12.45 using permutation). We attribute this to the combination of extreme logACR values with rare heterozygous genotypes that arise in many permutations of the data. The trend test is robust to this effect, but it risks missing associations with large dominant components.

**Table 1:** Genome-wide significance (0.05) thresholds for association mapping test statistics

		HDL	SBP	GLU	TG	log(ACR)
Simulation	Trendtest	5.37	5.17	5.53	5.51	8.00
	ANOVA	5.40	5.05	5.44	5.81	14.62
	EMMA	5.31	5.16	5.50	5.46	8.01
Permutation	Trendtest	5.46	5.29	5.51	5.74	7.79
	ANOVA	5.40	5.16	5.10	5.53	12.45
	EMMA	5.37	5.23	5.41	5.66	7.76

HDL, high-density lipoprotein; SBP, systolic blood pressure; GLU, glucose, TG, triglyceride; log(ACR), log-transformed albumin-to-creatinine ratio; ANOVA, analysis of variance.

### QTL mapping

**Figure 3:**
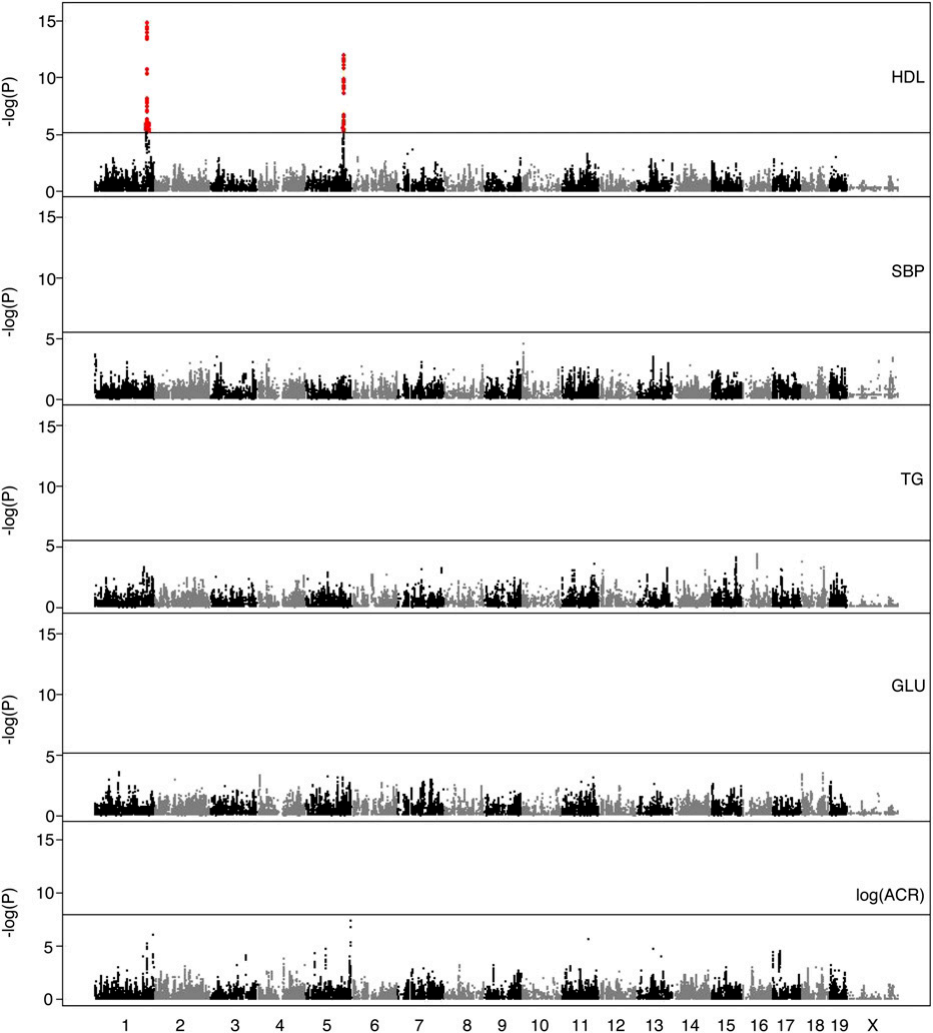
GWA mapping with mixed model analysis (EMMA) over 44,428 SNPs for traits HDL, SBP, TG, GLU, and the log-transformed ACR.

We performed GWA mapping using each of three test statistics, a simple trend test, ANOVA, and a trend test within a mixed model that accounts for family structure (EMMA). Results obtained using these tests across all five traits examined here were highly concordant (Figure 3 and Figure S4, A and B). The most striking findings were two highly significant loci associated with HDL. There was an association with SBP on proximal Chromosome 10 at 7 Mb that exceeded the genome-wide 0.05 thresholds for the simple trend and ANOVA tests, but fell below the threshold for the EMMA test. The logACR trait was the most variable of the five traits, although still highly concordant across test statistics. For this trait, we observed loci on Chromosome 5 at 147 Mb and Chromosome 11 at 88 Mb that exceeded the 0.05 threshold for either simple trend test or the ANOVA test. There were a number of subthreshold loci, in particular for logACR, that stood out from the background and may be of interest.

The strong associations for HDL on Chromosomes 1 and 5 (Figure S5) were also significantly associated with total cholesterol levels (not shown). The Chromosome 1 locus at 173 Mb spans a region encompassing 16 genes (Figure S6A). Numerous mouse crosses have linked HDL to this region and *Apoa2* has been identified as the gene underlying the QTL (Wang *et al.* 2004). In fact, rs8258226, which is believed to be the causative SNP in *Apoa2*, is on the Mouse Diversity Array and our NMRI animals are polymorphic at this SNP. As in the study by Wang *et al.*, the T allele (Valine at position 61) was associated with high HDL-C levels compared with the C allele (alanine at position 61). On Chromosome 5, the region of strongly associated SNPs contains 7 genes, including *Scarb1* (Figure S6B). This region has also been linked to HDL in multiple crosses, and *Scarb1* (encoding SRB1) was identified as the primary candidate gene (Su *et al.* 2010).

**Figure 4:**
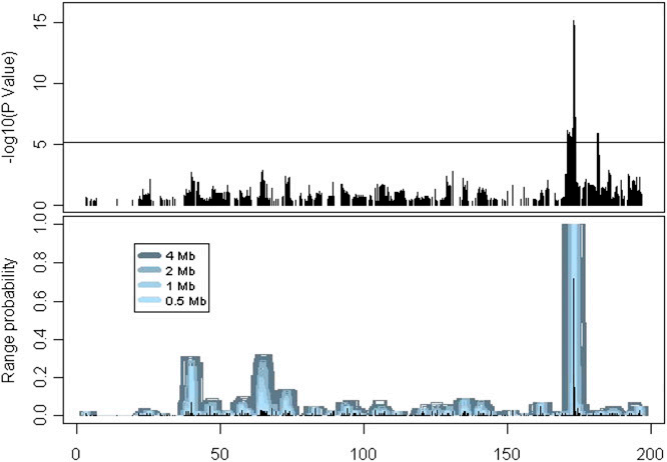
(Top panel) Detail of −log(P) statistics (EMMA) on distal Chromosome 1. (Bottom panel) Detail of bootstrap resampling statistics for HDL on Chromsome 1. Height of bar indicates how frequently a locus is included in a forward stepwise selection multilocus model.

In addition to the main peak at 173Mb on Chromosome 1, we observed a second distinct and significant peak at 181 Mb (Figure 4). This region has also been associated with HDL in mouse crosses that are not segregating *Apoa2* (R. Korstanje, personal observation and James Cheverud, personal communication). The peak SNPs at 173 Mb (rs31551271) and 181 Mb (rs30672856) were correlated (r = 0.36), although they are not in the same LD block (Figure S7). To establish whether these represent independent associations we repeated the genome scan analysis using EMMA with the peak SNP markers from Chromosome 1 and Chromosome 5 (rs36333480) as covariates. No new QTL were identified and the support for an independent QTL at 181Mb was reduced to a level consistent with chance (Figure S7). We also fit multilocus regression models with different combinations of the three QTL for HDL (Table S1). The Chromosome 1 locus at 181 Mb explains 10% of the total variance in HDL when considered on its own. However, after adjusting for the Chromosome 1 locus at 173 Mb, the QTL at 181 Mb accounts for only 2.5% of total variance. With the addition of the Chromosome 5 locus, this contribution decreases further, to 0.3% of total variance. Thus, the Chromosome 1 locus at 181 Mb may represent a spurious association in which the combined effects of two loci with genuine associations combine to create a “ghost” signal.

The absence of significant QTL for traits other than HDL is somewhat surprising. However, the distribution of *P*-values for these traits indicates that there may be an excess of small *P*-values, in particular for logACR (Figure S3). We identified the five strongest associations for each of the traits and compared them with published QTL for the same traits (Table 2). Almost all have been previously reported in mouse or rat linkage analyses. Thus, it is possible that these represent genuine signals that fall below the detection threshold of our study design.

**Table 2:** Top five peaks for the five traits and comparison with previously mapped QTL

Trait	Chr	Position (bp)	*P*-Value	QTL (Candidate Gene)	Reference
HDL	1	173,155,512	8.0 × 10^−16^	(*Apoa2*)	Wang *et al.* 2004
	5	125,530,593	6.0 × 10^−12^	(*Scarb1*)	Su *et al.* 2010
	1	181,672,702	8.4 × 10^−7^		
	7	52,328,410	2.4 × 10^−4^		
	11	86,772,383	5.4 × 10^−4^	B6 x C3H	Su *et al.* 2009
SBP	10	7,151,309	2.1 × 10^−5^	SBH x SBN (rat)	Yagil and Yagil 1998
	1	5,305,996	2.0 × 10^−4^		
	13	57,444,015	3.7 × 10^−4^		
	3	23,806,561	2.4 × 10^−4^	LH x LN (rat)	Sassard *et al.* 1997
	X	149,326,707	3.9 × 10^−4^		
TG	16	50,610,771	2.3 × 10^−5^	D1 × D2	Stylianou *et al.* 2008
	15	84,041,657	7.8 × 10^−5^	MRL/lpr x BALB/c	Gu *et al.* 1999
	18	4,740,277	1.8 × 10^−4^	—	
	11	107,124,592	3.1 × 10^−4^	B6 x D2	Colinayo *et al.* 2003
	1	164,192,866	3.9 × 10^−4^	B6 x RR	Suto *et al.* 2004
					NZO x NON	Su *et al.* 2009
GLU	1	83,642,895	2.6 × 10^−4^	B6 x 129	Kido *et al.* 2000
	18	73,383,043	3.9 × 10^−4^	B6 x CAST	Smith 2002
	18	4,707,155	4.4 × 10^−4^		
	4	8,691,086	5.5 × 10^−4^	B6 x D2	Togawa *et al.* 2006
	5	73,493,320	7.3 × 10^−4^		Chung *et al.* 1997
log(ACR)	5	147,841,950	4.6 × 10^−8^	MWF x LEW (rat)	Schulz *et al.* 2002
	1	194,419,611	9.3 × 10^−7^	B6 x NZM	Morel *et al.* 1994
	11	88,323,710	2.6 × 10^−6^	SS x SHR (rat)	Poyan Mehr *et al.* 2003
	1	174,219,650	6.7 × 10^−6^	B6 x NZM	Morel *et al.* 1994
	5	68,016,195	1.9 × 10^−5^	B6 x D2	Sheehan *et al.* 2007

QTL, quantitative trait loci; HDL, high-density lipoprotein; SBP, systolic blood pressure; GLU, glucose, TG, triglyceride; log(ACR), log-transformed albumin-to-creatinine ratio.

We conducted multilocus genome-wide scans using forward stepwise variable selection on bootstrapped samples. The QTL on Chromosome 1 at 173 Mb and Chromosome 5 at 126 Mb were included in 100% of resampled models but the QTL on Chromosome 1 at 181 Mb was never included as an independent QTL in the multi-locus analysis (Figure 4). The SBP QTL on proximal Chromosome 10 was represented in a substantial number (RMIP = 0.56) of the bootstrap samples. The TG QTL on Chromosome 16 at 51 Mb and two QTL for logACR (Chromosome 5 at 68 Mb and Chromosome 11 at 88 Mb) all had RMIP > 0.3.

## Discussion

We carried out a GWA study to discover and localize QTL in an outbred population sample of NMRI mice. Many commercially available outbred populations are not well suited for genetic mapping because of extensive inbreeding or other deficiencies. However, a number of these populations have high MAFs, low LD, and limited population structure, which makes them suitable for association mapping (Yalcin *et al.* 2010). Our NMRI mapping population satisfied these criteria and detected two significant QTL for HDL, demonstrating that loci with large effect sizes are readily detectable and can be mapped with high resolution. However, the sample size of 288 mice lacked the statistical power to detect alleles with small effects, leading to a number of suggestive associations. Most of the top scoring suggestive associations in our study replicate previously reported QTL. Failure to identify new loci suggests that the NMRI population carries mostly the same allelic variants characterized in complex traits analyses using classical inbred mouse strains.

Association mapping with outbred mouse populations is similar to human GWA studies in many respects, including the use of high-density SNP genotyping and genome-wide SNP-based association testing. We detected minimal population structure and observed rapid decay of LD within our NMRI population. However, the rate of LD decay is less than in wild-caught mice and human populations. Whereas there is no significant LD between markers more than 2 Mb apart in wild-caught mice (Laurie *et al.* 2007) and 0.5 Mb apart in humans (Dawson *et al.* 2002), the distance is approx 10 Mb in NMRI mice. As with human GWA studies, we assumed no previous knowledge of ancestral haplotypes in the founder population, which precludes inference-based linkage analysis methods. Our study differs from a human GWA in that the environment and phenotyping are tightly controlled and the population size is much smaller than is typical in human studies.

We identified significant association peaks for only one trait, HDL. We investigated the 5 peaks with the smallest *P*-value for each trait to see whether these non-significant peaks could be true associations. Nearly all were previously identified in mouse or rat linkage analyses. Concordance with known QTL suggests that these GWA peaks could be true-positive associations and that we could use the NMRI results to help identify candidate genes for the published QTL. For example, HDL was associated with a locus on Chromosome 11 (between 79.87 and 96.67 Mb) that includes 316 genes. A cross between C57BL/6J and C3H/HeJ mice previously linked HDL to this locus with a 95% CI between 57.00 and 88.50 Mb (including 854 genes). Assuming the same causal gene for the GWA and the QTL cross, we can narrow the search for candidate genes to the overlapping region from 79.87 to 88.50 Mb containing 177 genes. We can use high-density SNP data (Yang *et al.* 2011) to exclude regions that are identical-by-descent between C57BL/6J and C3H/HeJ mice based on the assumption that regions with no genetic variation between the two strains are unlikely to contain the QTL gene. In this way we eliminate 100 candidate genes. Of the 77 genes left in the interval, the gene encoding the hepatocyte nuclear factor 1β (*Hnf1b*) is a strong candidate because genetic deletion specifically in hepatocytes and bile duct cells significantly raises plasma cholesterol levels (Coffinier *et al.* 2002). Additional work will be required to experimentally validate candidate genes for these loci.

We expected the NMRI population to yield more significant QTL and to provide greater mapping resolution than we observed. Considering ancestry of NMRI, which originate from Swiss mice from Lausanne, provides a possible explanation. The mice were moved to the United States in 1926, transferred to the NIH in 1937, and subsequently inbred for 51 generations. The mice later moved to the Naval Medical Research Institute (and obtained the name NMRI), to the Zentral Institut fur Verzuchstierzucht in Hannover, and finally to Taconic. The bottleneck at the NIH likely reduced the genetic variation greatly. Most existing outbred populations, including NMRI, were derived from classical laboratory inbred strains and as such will have significant blind spots and limited haplotype diversity (Yang *et al.* 2011). Limited genetic diversity and uneven distribution of polymorphic loci may have limited the success of our mapping efforts using the NMRI population.

Spurious QTL detection in GWA studies can occur for a variety of reasons (Dickson *et al.* 2010). Multilocus QTL modeling can be useful to evaluate the robustness of QTL detected. We identified two association peaks for HDL on Chromosome 1 using single marker analysis but only one QTL at 173 Mb was supported in the multilocus analysis, which suggests that the second QTL may represent a spurious finding. Yalcin *et al.* (2010) reported a significant association for HDL at the *Apoa2* locus. They also report secondary association signals at 173.6Mb and 173.7Mb, substantially proximal to our locus at 181Mb. This region of Chromosome 1 has a complex LD structure in our NMRI sample (Figure 4) and among mouse inbred strains (Petkov *et al.* 2005). As a result it is difficult to determine if any of the observed associations distal to *Apoa2* are genuine or spurious.

Multiple testing presents a statistical challenge for both mouse and human GWA studies. The Bonferroni correction widely applied to human GWA analyses imposes a substantial multiple testing penalty, effectively limiting the ability to detect loci with small effects to reduce the detection of spurious loci. In our analysis, we used a novel permutation technique to calculate significance thresholds through simulation. Although permutation tests have been shown to be potentially misleading in the presence of family structure (Cheng *et al.* 2010), our simulation strategy accounts for population structure and for non-normal trait distributions. This permutation method could also be applied to human GWA studies. Although thresholds obtained with the simulation method did not differ substantially from those obtained with free permutation in this study, the two approaches would likely differ when applied to populations with more pronounced genetic structure. Therefore, we hesitate to recommend free permutation without a deeper investigation of the issues.

Precision of QTL mapping is not well understood in human GWA. We used a simulation approach to estimate mapping precision in our NMRI population and established that we can localize a QTL with large effect size to within 1.34 Mb of the greatest association peak. This result represents a genome-wide average and there may substantial local variation in mapping precision. This approach could be applied over a range of QTL effect sizes in any genotyped population sample including human GWA studies. Our assessment of mapping precision is tailored to the particular QTL found in this study. QTL of smaller effect size or in the context of a complex genetic background may be mapped with less precision.

This study demonstrates that GWA analysis can be successfully applied to outbred mice populations to identify genetic variants underlying complex traits. GWA studies can complement classical linkage analyses using inbred mouse crosses to refine QTL and identify causal genes, and future studies using outbred populations with greater genetic diversity should be even more powerful for identifying causal genes. However, the promise of currently available commercial outbred populations is limited by low genetic diversity and the requirement for sample sizes substantially larger than the approximately 00 animals used in this study.

## Supplementary Material

Supporting Information
