# Supplementary material for: Genome-Wide Association Mapping of Quantitative Traits in Outbred Mice
Source: G3 (Bethesda). 2012 Feb 1;2(2):167–74. doi: 10.1534/g3.111.001792 (PMC3284324; doi:10.1534/g3.111.001792)
Supplement: Supporting Information [file supp_2_2_167__index.html]

Supporting Information 

# Genome-Wide Association Mapping of Quantitative Traits in Outbred Mice

## Supporting Information for Zhang *et al.*, 2012

**Files in this Data Supplement:**

- Supporting Information - Figures S1-S7 and Table S1 (PDF, 1.9 MB)
- Figure S1 - Hierarchical cluster of kinship matrix for the NMRI population (PDF, 585 KB)
- Figure S2 - (A) Linkage disequilibrium on Chromosome 1 in the NMRI mapping population. (B) Close-up of the 170-185 Mb region. (PDF, 690 KB)
- Figure S3 - The distribution of the trait values (PDF, 47 KB)
- Figure S4 - Genome wide association mapping with simple linear trend test (A) and ANOVA test (B) (PDF, 385 KB)
- Figure S5 - Allele effects estimated at peak SNPs for the HDL association scan (PDF, 43 KB)
- Figure S6 - (A) Detail of the genome-wide association scan for HDL spanning significant (P<10-3) SNPs in the region around 173 Mb on Chromosome 1. (B) Detail of the genome-wide association scan for HDL spanning significant (P<10-3) SNPs in the region around 125 Mb on Chromosome 5. (PDF, 117 KB)
- Figure S7 - Detail of association scan for HDL showing -log(P) statistics for individual SNPs in the region 170-185 Mb on Chromosome 1 (A). Detail of the same region showing -log(P) statistics when peak SNPs on Chr1 and Chr5 are included as covariates in EMMA (B). (PDF, 59 KB)
- Table S1 - Variance explained by QTL for HDL (PDF, 34 KB)
