# Supplementary material for: Genome-Wide Association Mapping of Quantitative Traits in Outbred Mice
Source: G3 (Bethesda). 2012 Feb 1;2(2):167–74. doi: 10.1534/g3.111.001792 (PMC3284324; doi:10.1534/g3.111.001792)
Supplement: Supporting Information [file supp_2.2.167_001792SI.pdf]

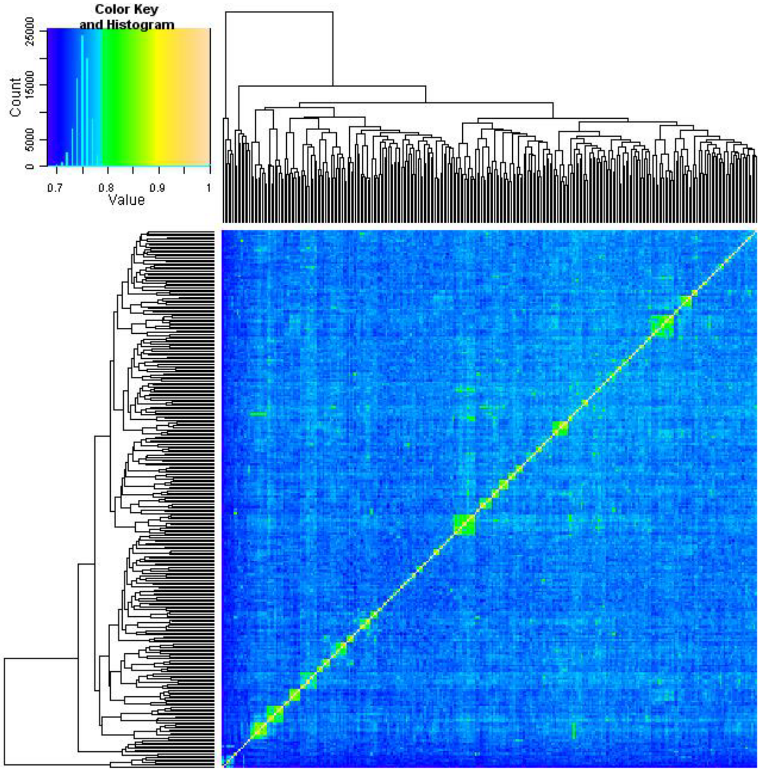

Figure S1 Hierarchical cluster of kinship matrix for the NMRI population.

A

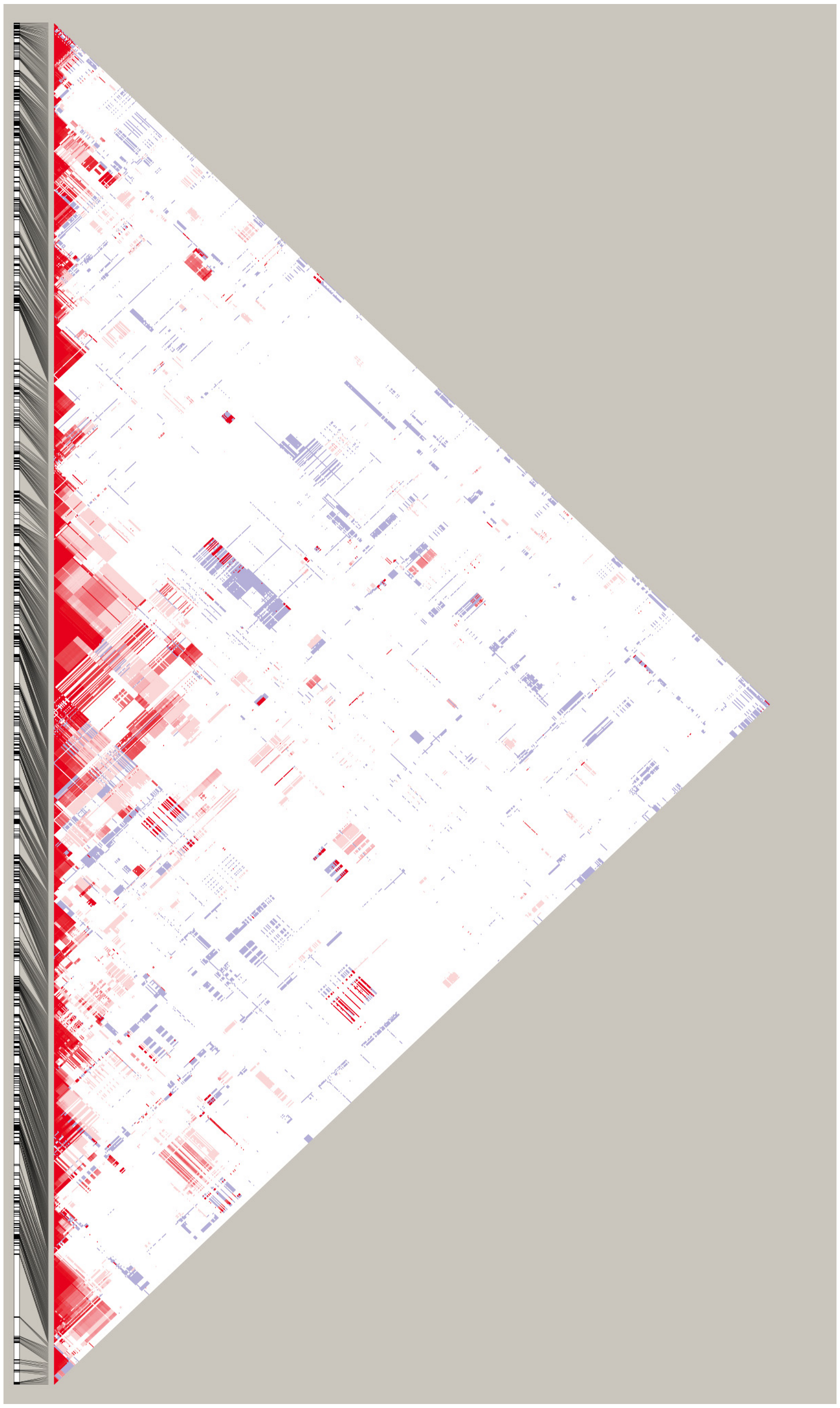

B

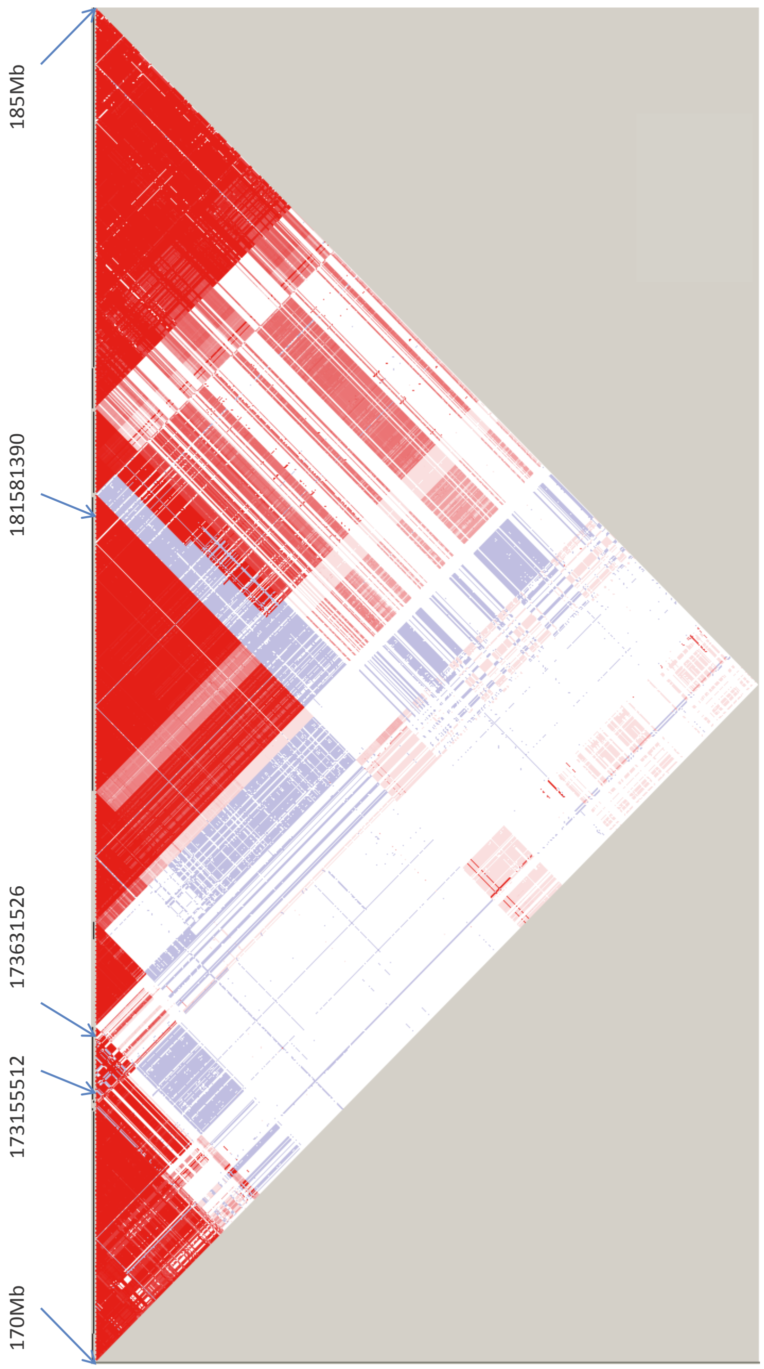

Figure S2 (A) Linkage disequilibrium on Chromosome 1 in the NMRI mapping population. The image was generated with Haploview. (B) Close-up of the 170-185 Mb region.

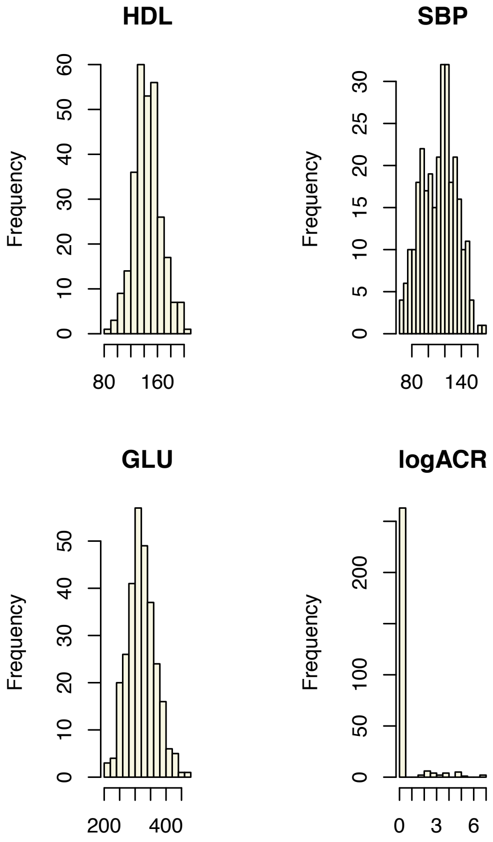

Figure S3 The distribution of the trait values.

A

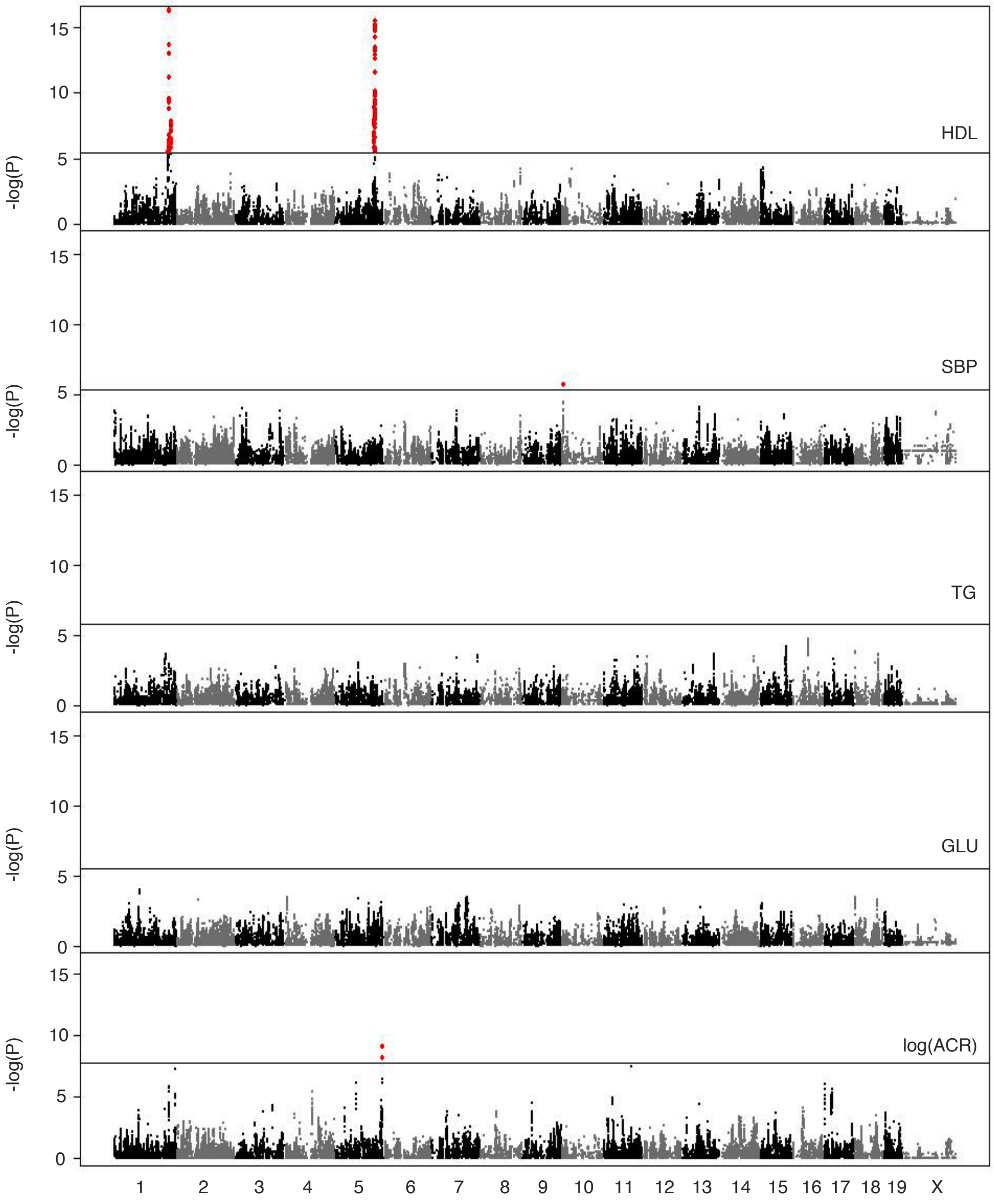

HDL
SBP
TG
GLU
log(ACR)
-log(P)
0
5
10
15
1
2
3
4
5
6
7
8
9
10
11
12
13
14
15
16
17
18
19
X

B

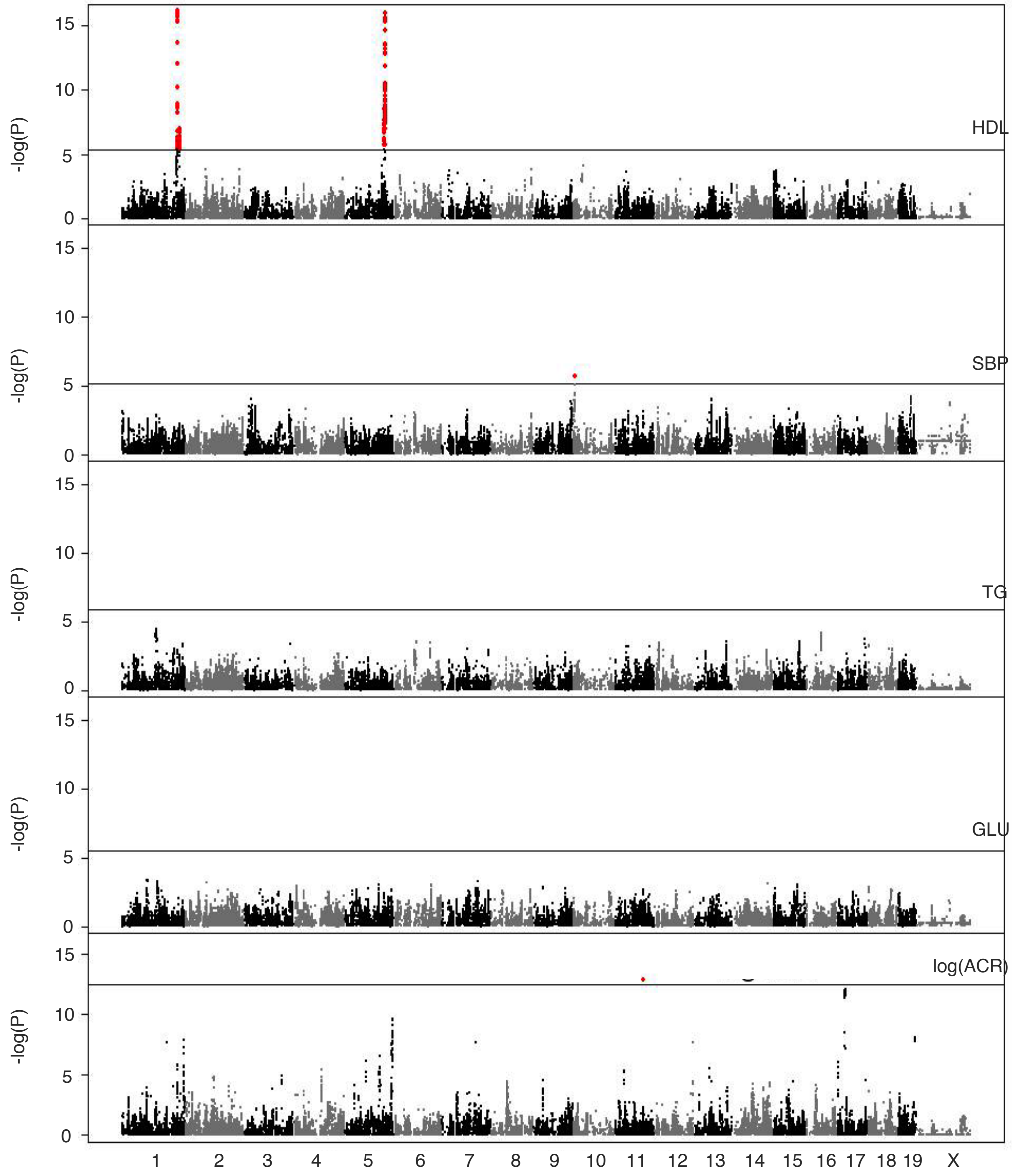

Figure S4 Genome wide association mapping with simple linear trend test (A) and ANOVA test (B)

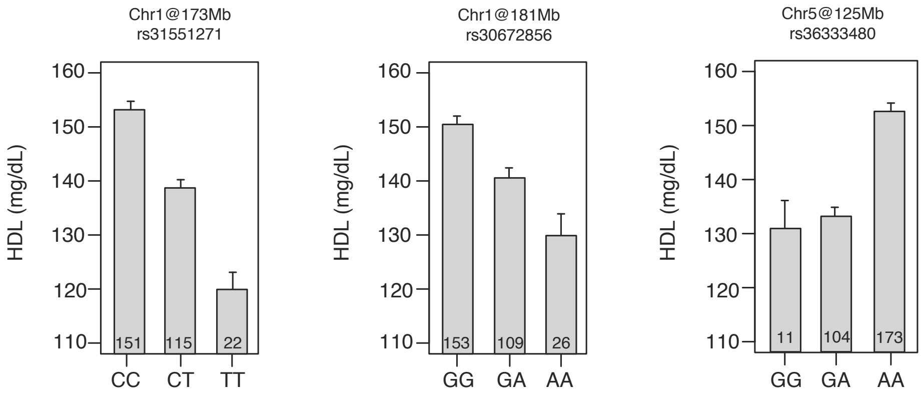

Figure S5 Allele effects estimated at peak SNPs for the HDL association scan.

A

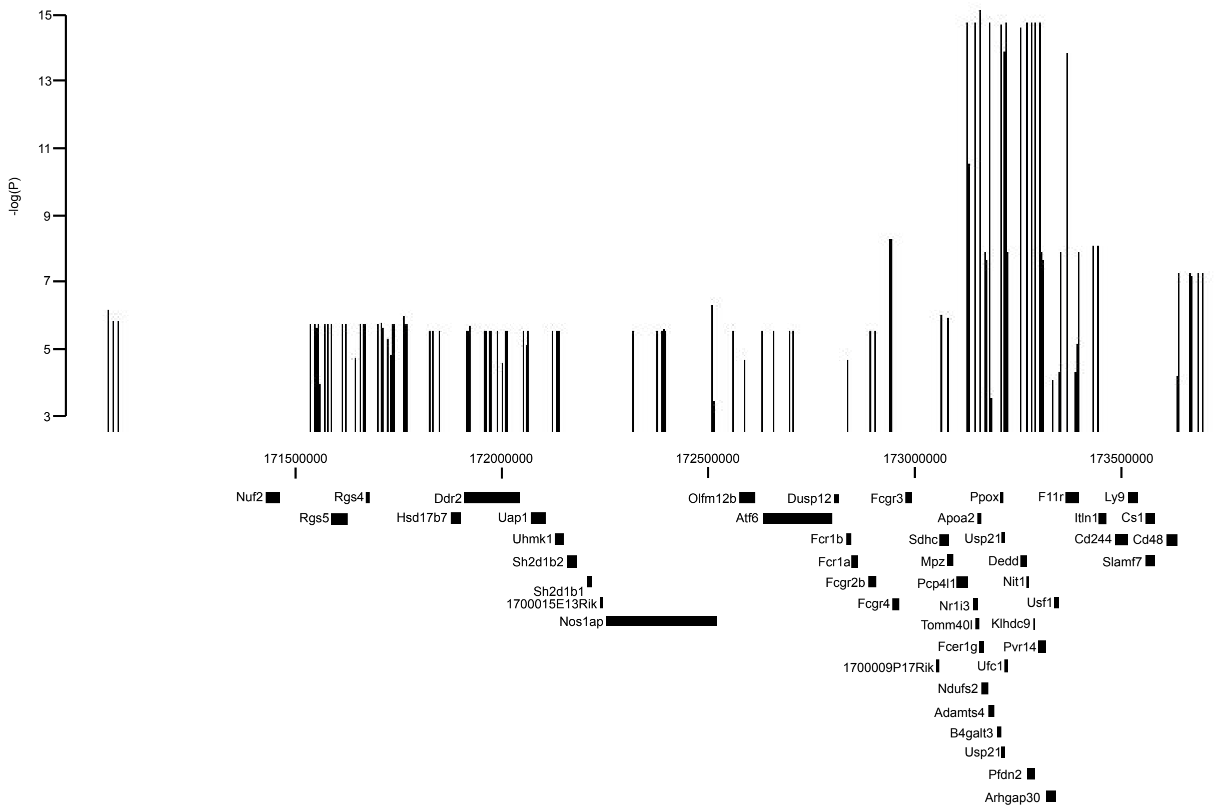

-log(P)
15
13
11
9
7
5
3
171500000
172000000
172500000
173000000
173500000
Nuf2
Rgs4
Rgs5
Ddr2
Hsd17b7
Uap1
Uhmk1
Sh2d1b2
Sh2d1b1
1700015E13Rik
Nos1ap
Olfm12b
Atf6
Dusp12
Fcr1b
Fcr1a
Fcgr2b
Fcgr4
Fcgr3
Apoa2
Sdhc
Mpz
Pcp4l1
Nr1i3
Tomm40l
Fcer1g
1700009P17Rik
Ndufs2
Adamts4
B4galt3
Usp21
Pfdn2
Arhgap30
Ppox
Usp21
Dedd
Nit1
Usf1
Klhdc9
Pvr14
Ufc1
F11r
Itln1
Cd244
Ly9
Cs1
Cd48
Slamf7

B

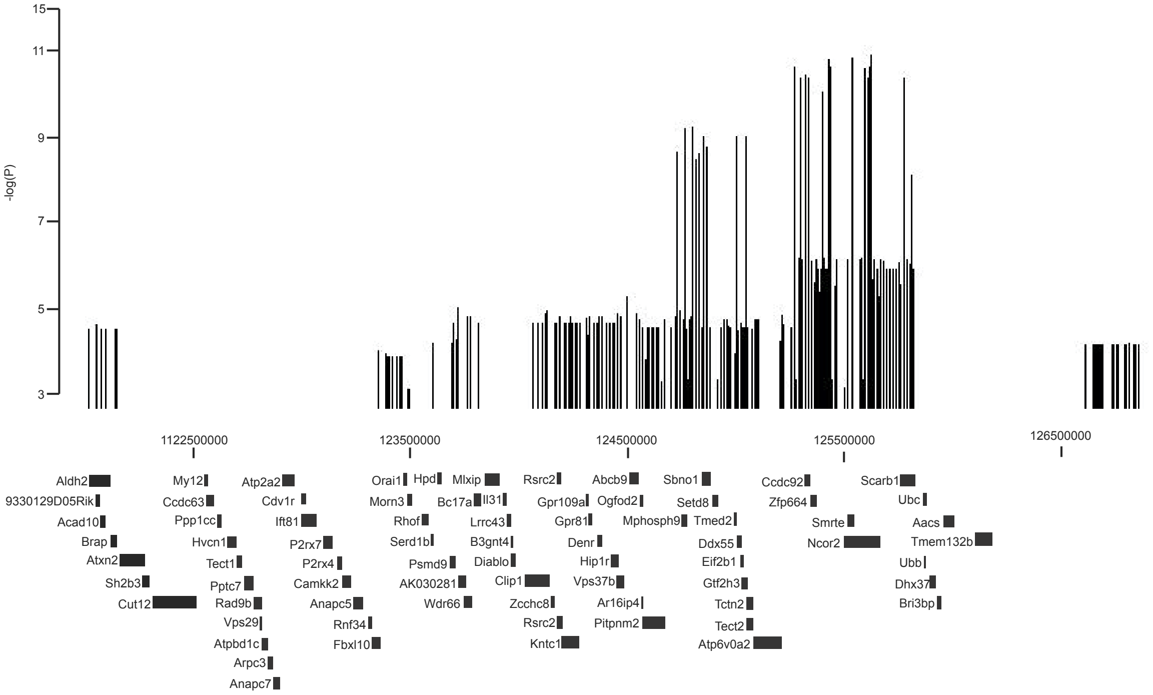

Figure S6 (A) Detail of the genome-wide association scan for HDL spanning significant ($P<10^{-3}$) SNPs in the region around 173 Mb on Chromosome 1. The -log(P) statistics are taken from the mixed model (EMMA). Annotated protein coding gene from UCSC are shown below for reference. (B) Detail of the genome-wide association scan for HDL spanning significant ($P<10^{-3}$) SNPs in the region around 125 Mb on Chromosome 5. The -log(P) statistics are taken from the mixed model (EMMA). Annotated protein coding gene from UCSC are shown below for reference.

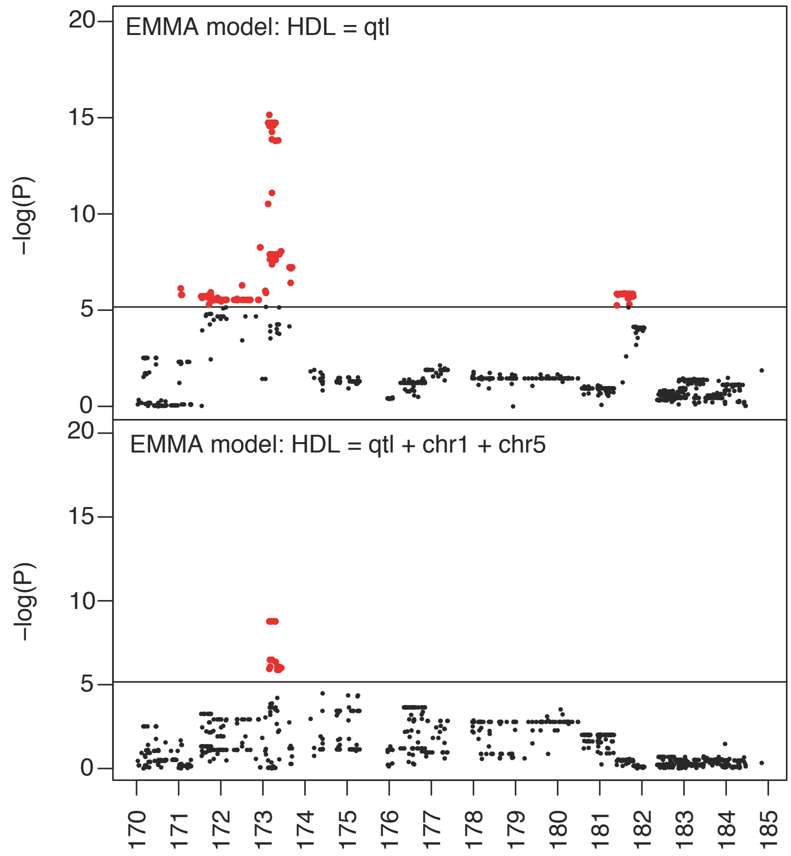

Figure S7 Detail of association scan for HDL showing -log(P) statistics for individual SNPs in the region 170-185 Mb on Chromosome 1 (A). Detail of the same region showing -log(P) statistics when peak SNPs on Chr1 and Chr5 are included as covariates in EMMA (B).

Table S1 Variance explained by QTL for HDL.

Model	%Var
Chr1@173Mb	23.7
Chr1@181Mb	10.6
Chr5@125Mb	20.9
Chr1@173 + Chr1@181	26.2
Chr1@173+Chr5@125	41.6
Chr1@181+Chr5@125	25.7
Chr1@173+Chr1@181+Chr5@125	41.9
