# Supplementary figures and images for: Genome-Wide Association Mapping of Quantitative Traits in Outbred Mice
Source: G3 (Bethesda). 2012 Feb 1;2(2):167–74. doi: 10.1534/g3.111.001792 (PMC3284324; doi:10.1534/g3.111.001792)

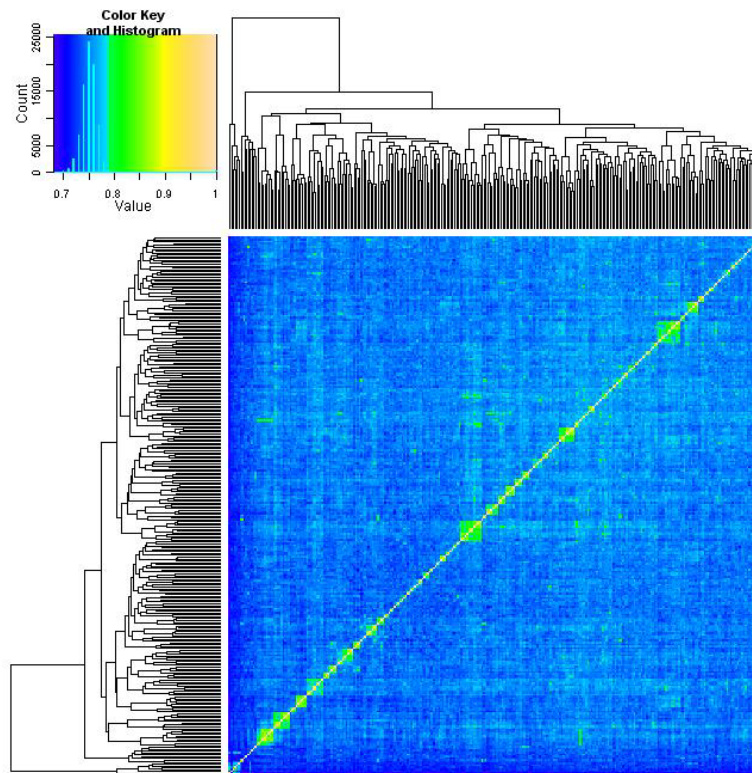

**Figure S1** Hierarchical cluster of kinship matrix for the NMRI population.

Supplement: Supporting Information [file supp_2.2.167_FigureS1.pdf]

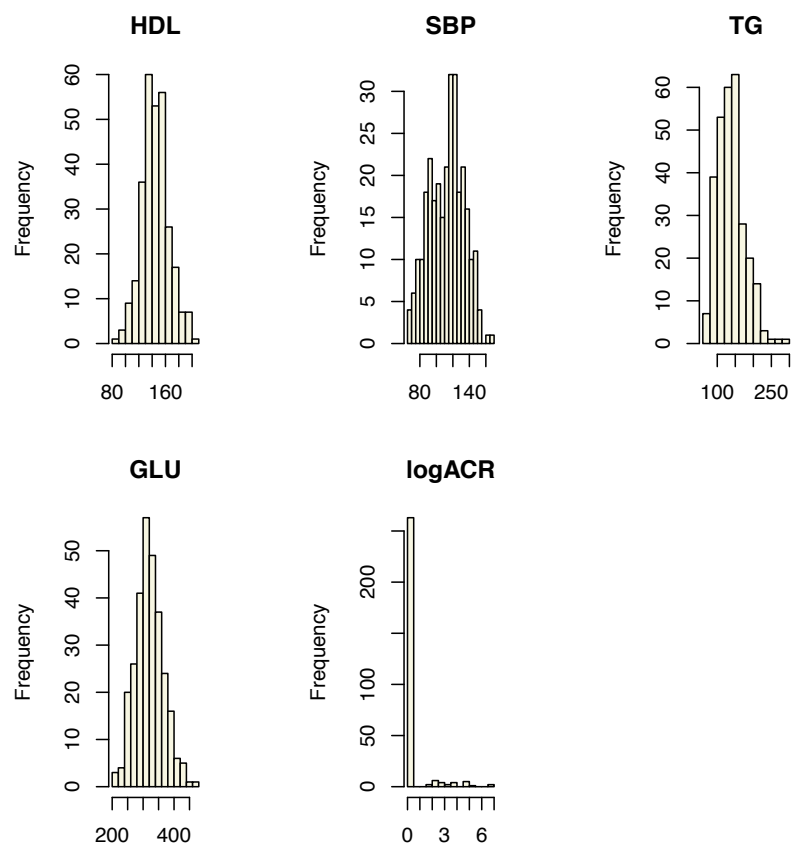

**Figure S3** The distribution of the trait values.

Supplement: Supporting Information [file supp_2.2.167_FigureS3.pdf]

A

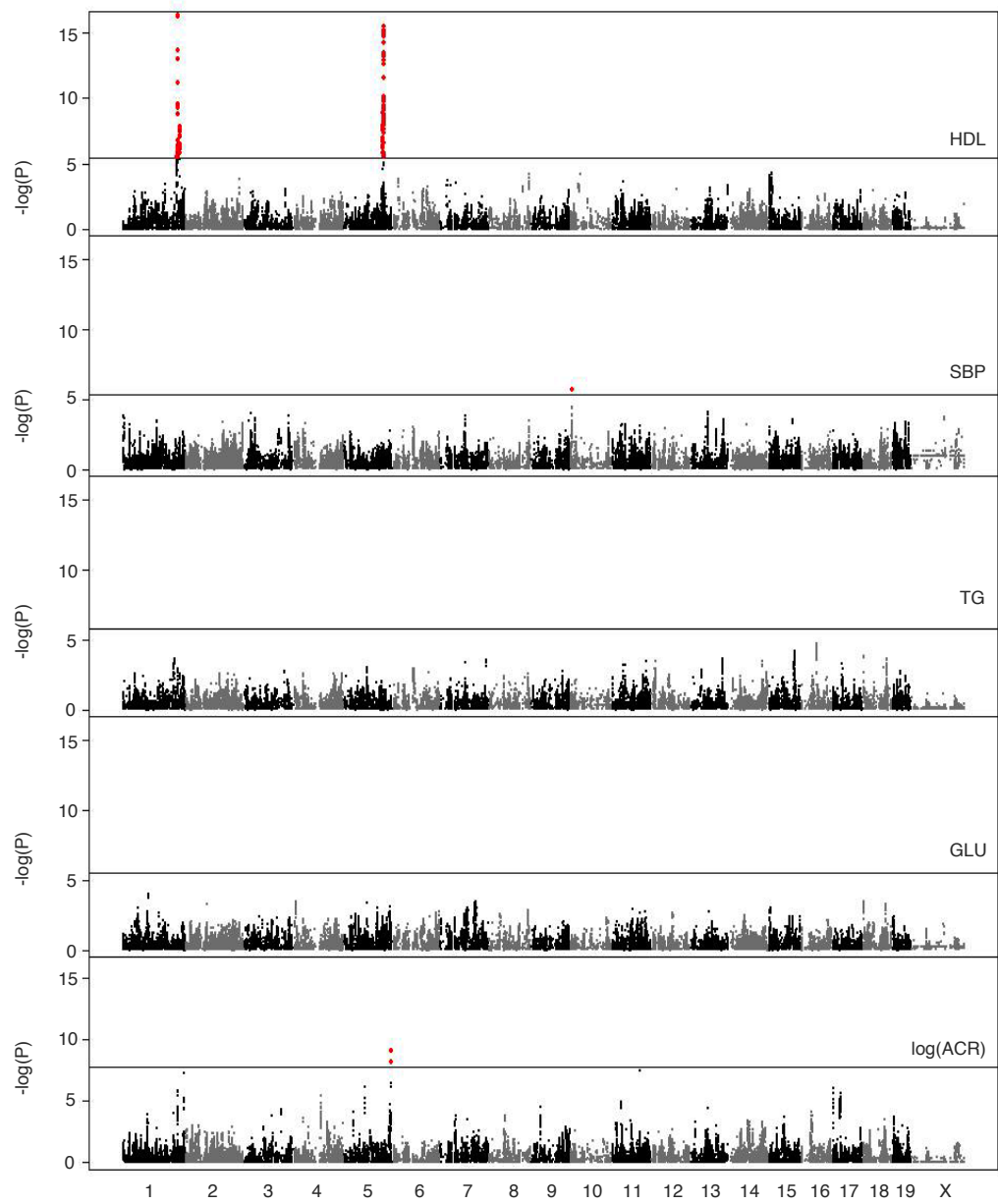

**B**

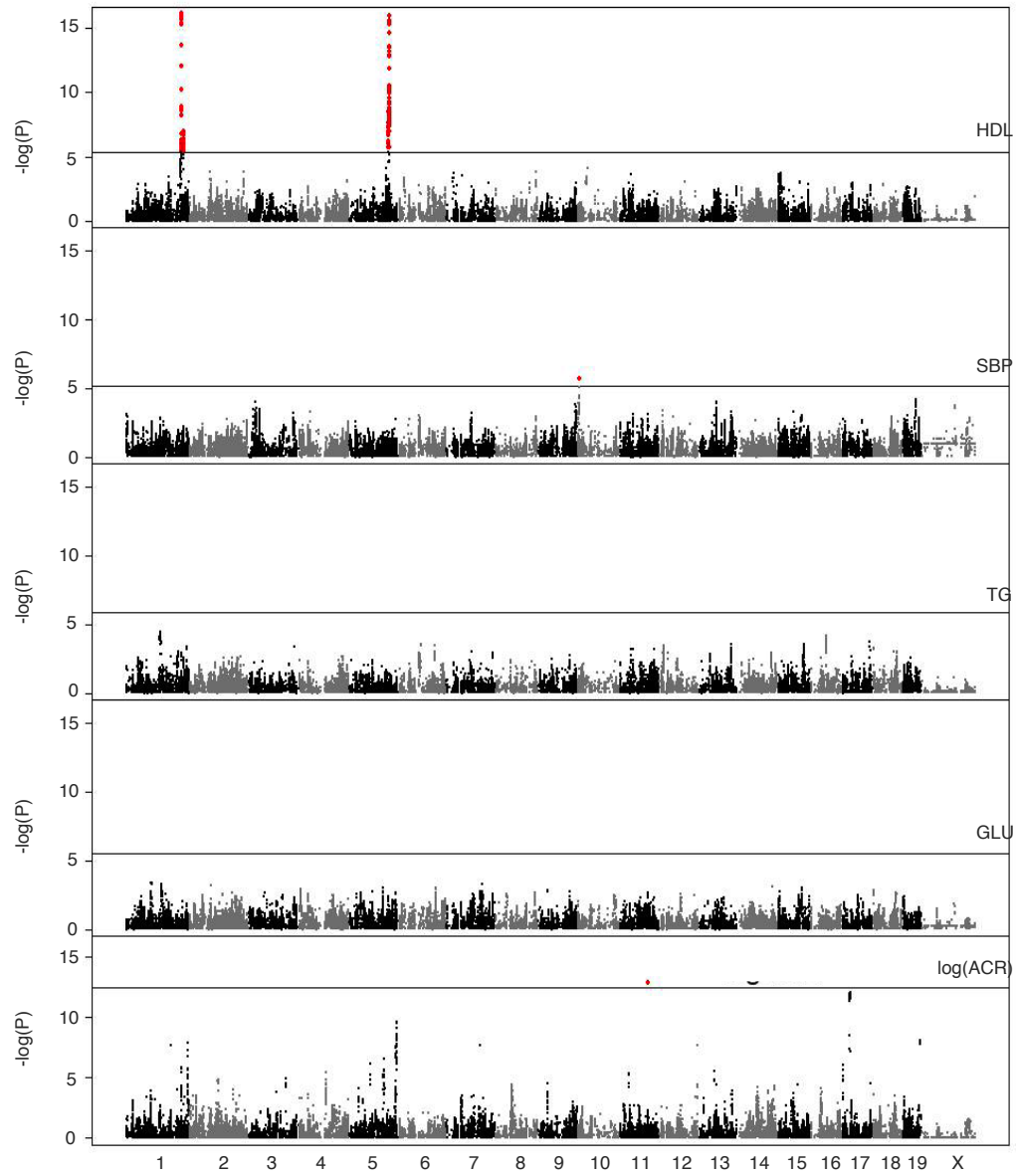

**Figure S4** Genome wide association mapping with simple linear trend test (A) and ANOVA test (B)

Supplement: Supporting Information [file supp_2.2.167_FigureS4.pdf]

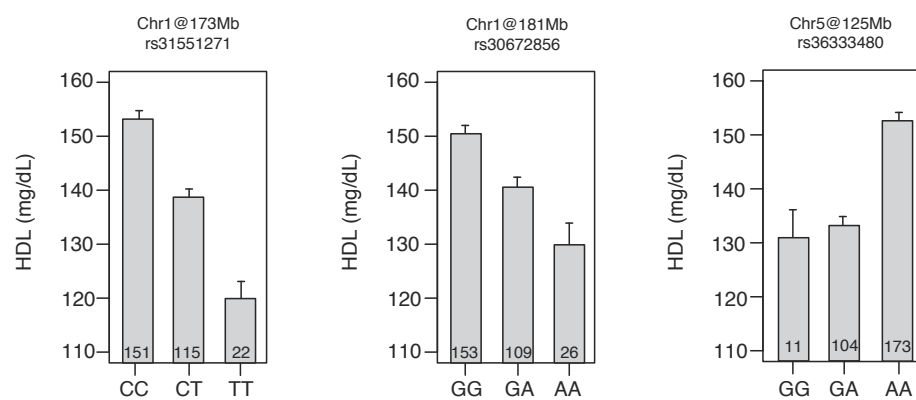

**Figure S5** Allele effects estimated at peak SNPs for the HDL association scan.

Supplement: Supporting Information [file supp_2.2.167_FigureS5.pdf]
