# Supplementary material for: Genome-Wide Association Mapping of Quantitative Traits in Outbred Mice
Source: G3 (Bethesda). 2012 Feb 1;2(2):167–74. doi: 10.1534/g3.111.001792 (PMC3284324; doi:10.1534/g3.111.001792)
Supplement: Supporting Information [file supp_2.2.167_FigureS2.pdf]

A

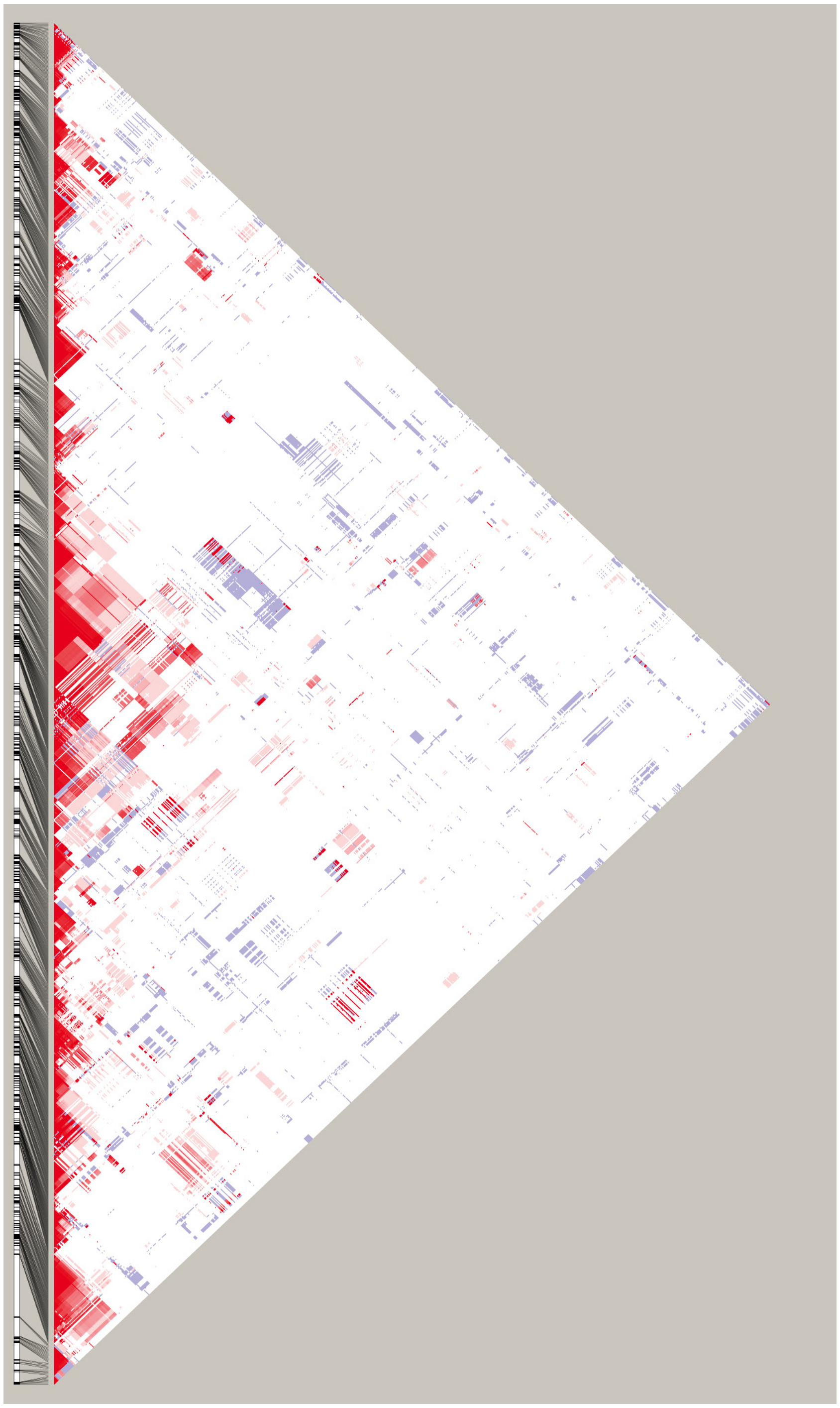

B

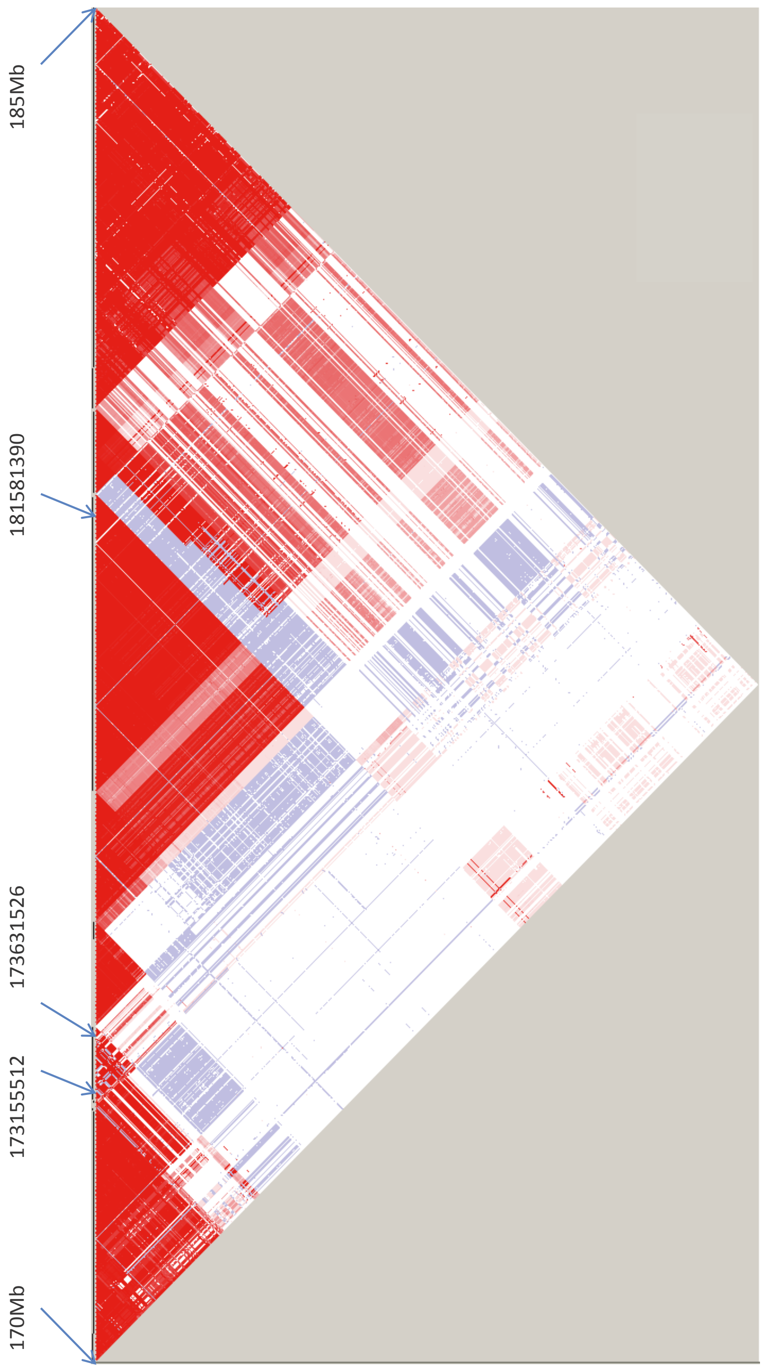

Figure S2 (A) Linkage disequilibrium on Chromosome 1 in the NMRI mapping population. The image was generated with Haploview. (B) Close-up of the 170-185 Mb region.
