# Supplementary material for: Genome-Wide Association Mapping of Quantitative Traits in Outbred Mice
Source: G3 (Bethesda). 2012 Feb 1;2(2):167–74. doi: 10.1534/g3.111.001792 (PMC3284324; doi:10.1534/g3.111.001792)
Supplement: Supporting Information [file supp_2.2.167_FigureS6.pdf]

A

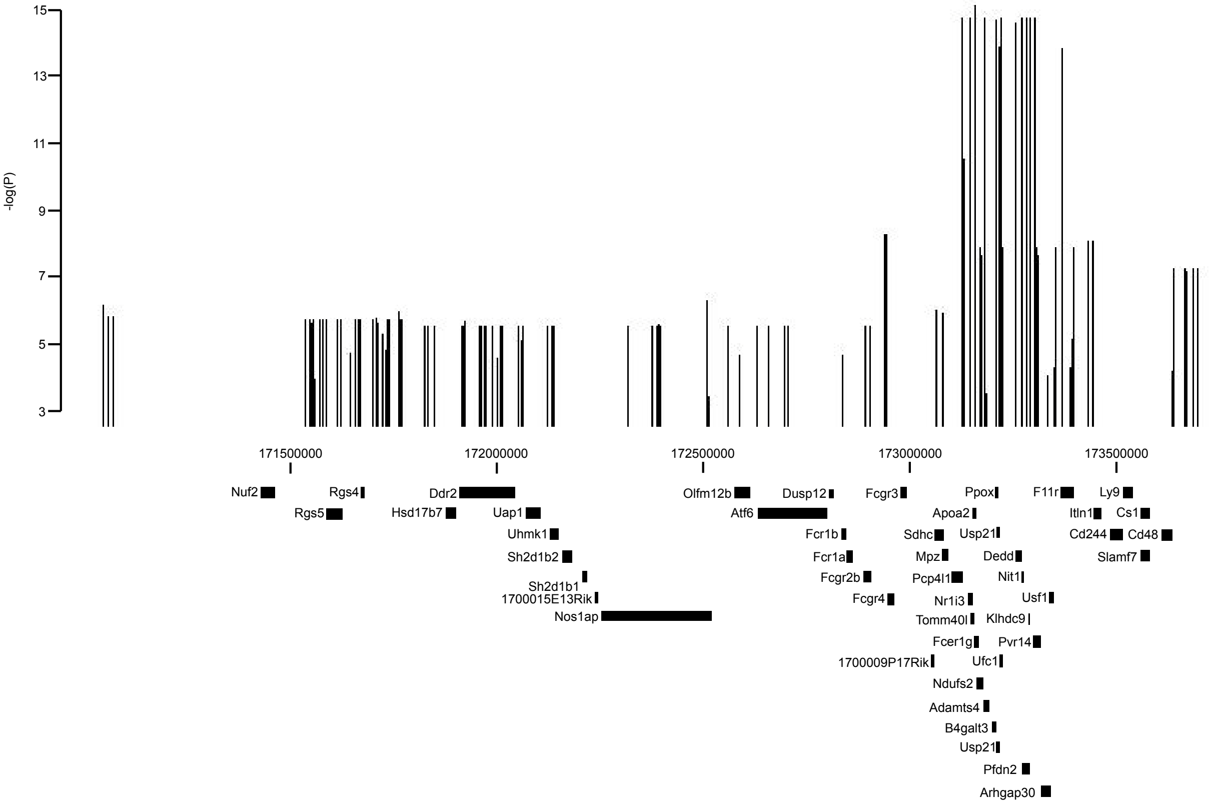

-log(P)
15
13
11
9
7
5
3
171500000
172000000
172500000
173000000
173500000
Nuf2
Rgs4
Rgs5
Ddr2
Hsd17b7
Uap1
Uhmk1
Sh2d1b2
Sh2d1b1
1700015E13Rik
Nos1ap
Olfm12b
Atf6
Dusp12
Fcr1b
Fcr1a
Fcgr2b
Fcgr4
Fcgr3
Apoa2
Sdhc
Mpz
Pcp4l1
Nr1i3
Tomm40l
Fcer1g
1700009P17Rik
Ndufs2
Adamts4
B4galt3
Usp21
Pfdn2
Arhgap30
Ppox
Usp21
Dedd
Nit1
Usf1
Klhdc9
Pvr14
Ufc1
F11r
Itln1
Cd244
Ly9
Cs1
Cd48
Slamf7

B

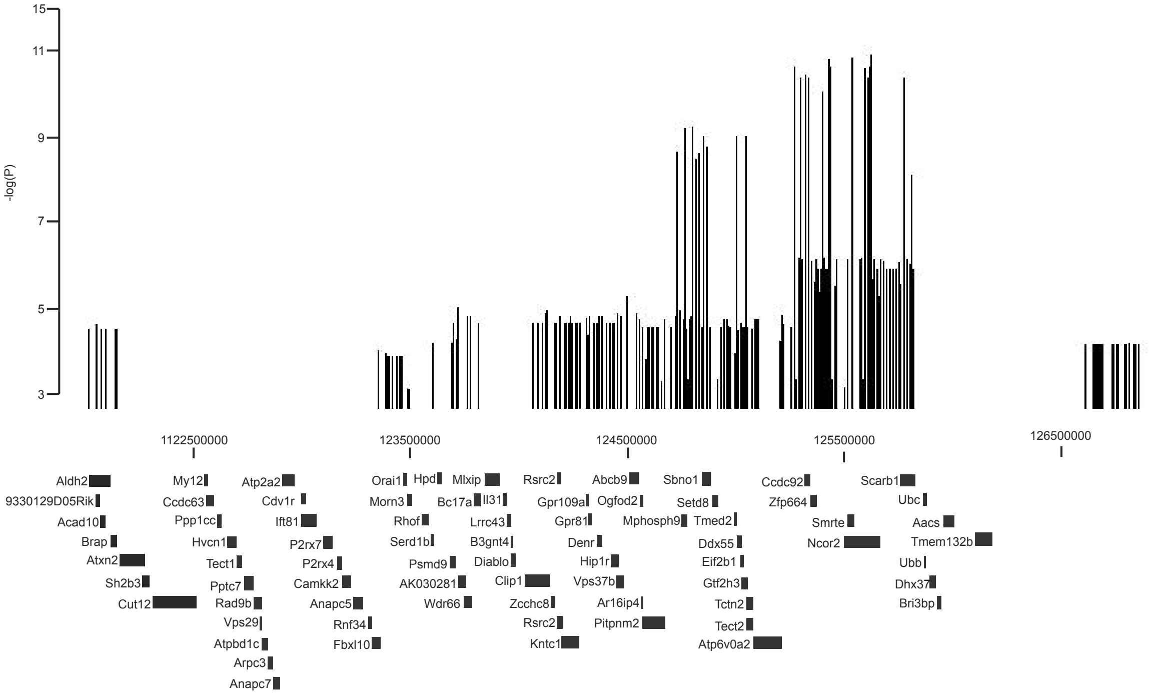

Figure S6 (A) Detail of the genome-wide association scan for HDL spanning significant ($P<10^{-3}$) SNPs in the region around 173 Mb on Chromosome 1. The -log(P) statistics are taken from the mixed model (EMMA). Annotated protein coding gene from UCSC are shown below for reference. (B) Detail of the genome-wide association scan for HDL spanning significant ($P<10^{-3}$) SNPs in the region around 125 Mb on Chromosome 5. The -log(P) statistics are taken from the mixed model (EMMA). Annotated protein coding gene from UCSC are shown below for reference.
