# Supplementary material for: Genome-Wide Association Mapping of Quantitative Traits in Outbred Mice
Source: G3 (Bethesda). 2012 Feb 1;2(2):167–74. doi: 10.1534/g3.111.001792 (PMC3284324; doi:10.1534/g3.111.001792)
Supplement: Supporting Information [file supp_2.2.167_FigureS7.pdf]

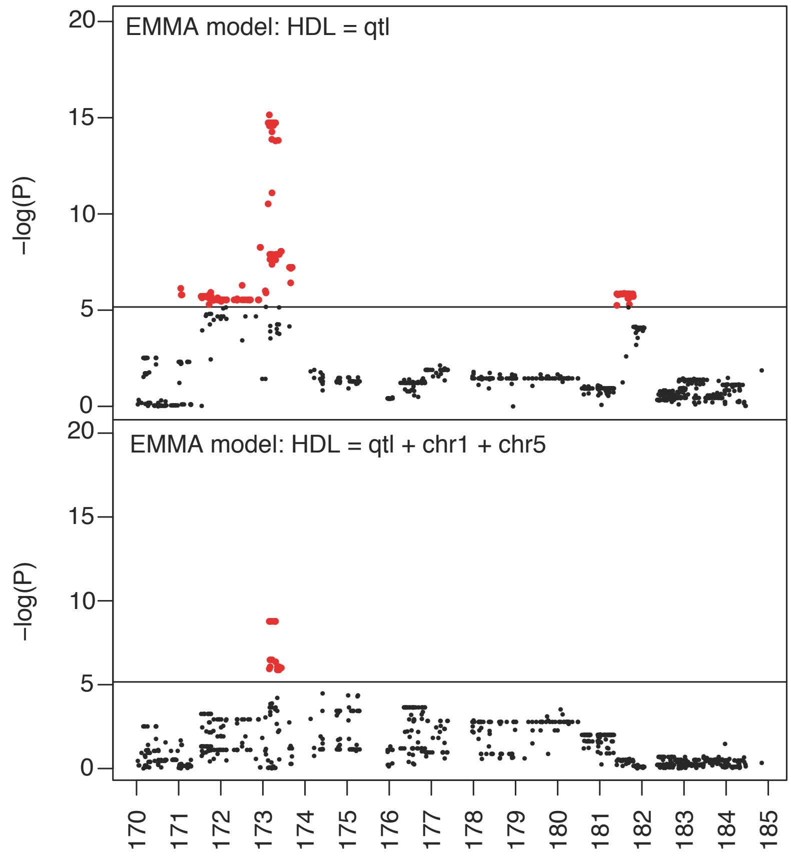

Figure S7 Detail of association scan for HDL showing -log(P) statistics for individual SNPs in the region 170-185 Mb on Chromosome 1 (A). Detail of the same region showing -log(P) statistics when peak SNPs on Chr1 and Chr5 are included as covariates in EMMA (B).
