# Supplementary material for: Genome-Wide Association Mapping of Quantitative Traits in Outbred Mice
Source: G3 (Bethesda). 2012 Feb 1;2(2):167–74. doi: 10.1534/g3.111.001792 (PMC3284324; doi:10.1534/g3.111.001792)
Supplement: Supporting Information [file supp_2.2.167_TableS1.pdf]

Table S1 Variance explained by QTL for HDL.

Model	%Var
Chr1@173Mb	23.7
Chr1@181Mb	10.6
Chr5@125Mb	20.9
Chr1@173 + Chr1@181	26.2
Chr1@173+Chr5@125	41.6
Chr1@181+Chr5@125	25.7
Chr1@173+Chr1@181+Chr5@125	41.9
